# Cell Confluence Modulates TRPV4 Channel Activity in Response to Hypoxia

**DOI:** 10.3390/biom12070954

**Published:** 2022-07-07

**Authors:** Solène Barbeau, Alexandre Joushomme, Yann Chappe, Guillaume Cardouat, Isabelle Baudrimont, Véronique Freund-Michel, Christelle Guibert, Roger Marthan, Patrick Berger, Pierre Vacher, Yann Percherancier, Jean-François Quignard, Thomas Ducret

**Affiliations:** 1 Centre de Recherche Cardio-Thoracique de Bordeaux, Univ. Bordeaux, U1045, F-33600 Pessac, France; solene.barbeau@u-bordeaux.fr (S.B.); guillaume.cardouat@u-bordeaux.fr (G.C.); isabelle.baudrimont@u-bordeaux.fr (I.B.); veronique.michel@u-bordeaux.fr (V.F.-M.); christelle.guibert@u-bordeaux.fr (C.G.); roger.marthan@u-bordeaux.fr (R.M.); patrick.berger@u-bordeaux.fr (P.B.); pierre.vacher@inserm.fr (P.V.); jean-francois.quignard@u-bordeaux.fr (J.-F.Q.); 2 INSERM (Institut National de la Santé Et de la Recherche Médicale), Centre de Recherche Cardio-Thoracique de Bordeaux, U1045, F-33600 Pessac, France; 3 Laboratoire de l’Intégration du Matériau au Système, UMR5518, Univ. Bordeaux, F-33400 Talence, France; alexandre.joushomme@u-bordeaux.fr (A.J.); yann.chappe@u-bordeaux.fr (Y.C.); yann.percherancier@u-bordeaux.fr (Y.P.); 4 CNRS (Centre National de la Recherche Scientifique), Laboratoire de L’integration du Matériau au Système, UMR5518, F-33400 Talence, France; 5 CHU (Centre Hospitalier Universitaire) Bordeaux, Service d’Exploration Fonctionnelle Respiratoire, F-33600 Pessac, France

**Keywords:** BRET, calcium, cell culture conditions, cell density, patch-clamp, stretch-activated channel, TRP channel

## Abstract

Transient receptor potential vanilloid 4 (TRPV4) is a polymodal Ca^2+^-permeable channel involved in various hypoxia-sensitive pathophysiological phenomena. Different tools are available to study channel activity, requiring cells to be cultured at specific optimal densities. In the present study, we examined if cell density may influence the effect of hypoxia on TRPV4 activity. Transiently TRPV4-transfected HEK293T cells were seeded at low or high densities corresponding to non-confluent or confluent cells, respectively, on the day of experiments, and cultured under in vitro normoxia or hypoxia. TRPV4-mediated cytosolic Ca^2+^ responses, single-channel currents, and Ca^2+^ influx through the channel were measured using Ca^2+^ imaging/microspectrofluorimetric assay, patch-clamp, and Bioluminescence Resonance Energy Transfer (BRET), respectively. TRPV4 plasma membrane translocation was studied using confocal microscopy, biotinylation of cell surface proteins, and BRET. Our results show that hypoxia exposure has a differential effect on TRPV4 activation depending on cell confluence. At low confluence levels, TRPV4 response is increased in hypoxia, whereas at high confluence levels, TRPV4 response is strongly inhibited, due to channel internalization. Thus, cell density appears to be a crucial parameter for TRPV4 channel activity.

## 1. Introduction

Transient receptor potential (TRP) channels constitute a superfamily of non-selective cationic channels that exhibit a common structure. They are composed of N- and C-terminal regions containing protein interaction motifs and six transmembrane domains (TM1-TM6). The putative ion conducting pore is located between the fifth and sixth TM domains. According to their activation stimuli and the presence of regulatory domains on the cytosolic N- and C-termini, the TRP superfamily is subdivided into seven main subfamilies: TRPC1-7 (Canonical), TRPV1-6 (Vanilloid), TRPM1-8 (Melastatin), TRPP1-4 (Polycystin), TRPML1-3 (Mucolipin), TRPA1 (Ankyrin), and TRPN (no mechanoreceptor potential C, also named NOMPC). Functional TRPs are composed of four homo- or heteromultimeric subunits.

Among TRPV channels, TRPV4 is a Ca^2+^-permeable channel whose expression in mammals is widespread throughout the body: ear, eye, skin, cardiovascular, digestive, immune, musculoskeletal, nervous, reproductive, and respiratory systems (for review see [1]). Due to its Ca^2+^ permeability (PCa/PNa ≈ 7 in HEK293 cells transiently transfected with wild type TRPV4, [2]), activation of TRPV4 induces an increase in cytosolic Ca^2+^ concentration ([Ca^2+^]_c_) which plays a key role in fundamental cellular processes such as apoptosis, contraction, migration, and proliferation in many cell types. Thus, owing to its ubiquitous expression and its role in mediating Ca^2+^ influx, it is now recognized that TRPV4 supports a great number of roles in various physiological and disease states [3].

A wide variety of stimuli, including physical (cell swelling and shear stress), thermal (moderate heat), and chemical (endogenous and exogenous ligands) stimuli such as the arachidonic acid metabolite epoxyeicosatrienoic acid and the synthetic agonist GSK-1016790A, in particular [4], activate TRPV4. In addition, other modulatory intermediates affect the channel function. Indeed, a range of phosphorylations or protein–protein interactions affect the sensitivity to gating stimuli or channel trafficking. For example, in TRPV4 transfected HEK293 cells, the serine/threonine protein kinases A and C (PKA and PKC) modulate the gating of the channel by cell swelling [5]. In the same cellular model (neurons), plasma membrane localization of TRPV4 is also modulated by the cytoskeletal protein kinase C and casein kinase substrate (PACSIN 3) [6], the ubiquitin ligase AIP4 [7], the ubiquitously expressed endoplasmic reticulum-associated protein OS-9 [8], the stromal interaction molecule 1 (STIM1) [9], or the phosphatidylinositol 3-kinase (PI3K) [10]. Moreover, several studies show a modulatory role of hypoxia on TRPV4 channel function, which occurs, not only in a variety of chronic lung diseases [11], but also in other organ systems and related diseases [12]. Indeed, TRPV4 activity is increased by hypoxic stimuli in rat pulmonary arterial smooth muscle cells [13,14,15], human umbilical vascular endothelial cells [16], rat hippocampal astrocytes [17], human SH-SY5Y neuroblastoma cell line [18], rat heart tissue-derived H9C2 cardiac myoblast cell line, and neonatal rat ventricle myocytes [19].

Whereas TRPV4 is obviously mechanosensitive [20], there is additional evidence that parameters such as confluence for adherent cells affect whole-cell mechanical behaviour, e.g., confluent fibroblastic cells were 50% stiffer than non-confluent ones [21]. Therefore, culture conditions under which the cell seeding is performed may modulate the functionality of the channel. Thus, in the present study, we investigated the influence of cell density on the effect of hypoxia on TRPV4 activity. We sought to compare TRPV4-induced cytosolic Ca^2+^ responses (Ca^2+^ imaging and microspectrofluorimetric assay) and single channel activity (patch-clamp technique in cell-attached configuration) in transiently TRPV4-transfected HEK293T cells seeded at low (non-confluent cells) and high (confluent cells) densities, and cultured under in vitro normoxia (21% O_2_) or hypoxia (1% O_2_) for 48 h. Note that in this study, the terms “normoxia” and “hypoxia” are used to distinguish between the various levels of oxygenation in the incubator. Thus, “normoxia” corresponds to atmospheric oxygen pressure, the commonly used oxygen pressure for cell cultures, but not to “tissue normoxia” also called “physioxia” which is characteristic of each tissue [22].

Direct Ca^2+^ influx through the channel was measured using an intramolecular Bioluminescence Resonance Energy Transfer (BRET) probe in which the TRPV4 channel was fused to a BRET sensor with high affinity for Ca^2+^ (Calflux-VTN) [23]. TRPV4 plasma membrane translocation was studied using confocal microscopy, cell surface biotinylation of proteins, and bystander BRET in which TRPV4 channel membrane density was monitored [24]. Our results show that hypoxia exposure has a differential effect on TRPV4 activation depending on cell confluence. At low confluence levels, hypoxia increases TRPV4 response, whereas at high confluence levels, TRPV4 response is strongly inhibited, due to channel internalization. Thus, cell density appears to be a crucial parameter for TRPV4 channel activity.

## 2. Materials and Methods

### 2.1. Cell Culture

**Figure 1:**
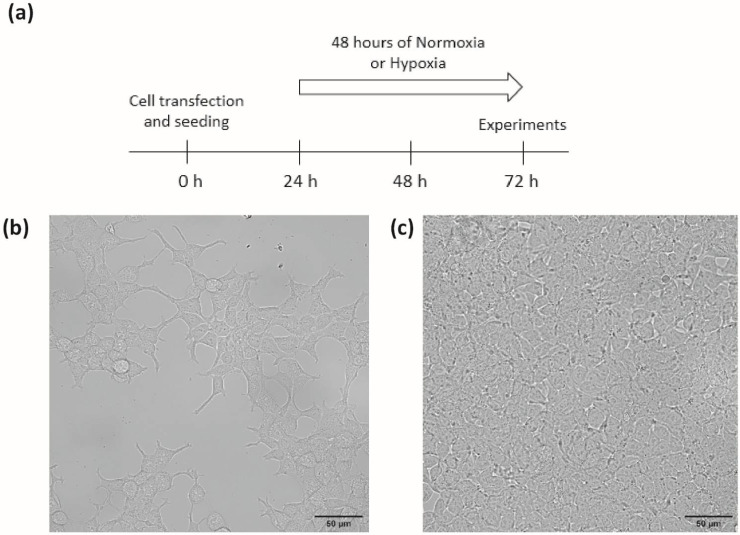
Cell culture conditions (**a**) timeline protocol for cell experiments, (**b**,**c**) representative phase-contrast fields of HEK293T cells seeded at (**b**) low (1 × 10^4^ cells/cm^2^) or (**c**) high (3 × 10^5^ cells/cm^2^) densities after 72 h of culture. Scale bar = 50 µm (magnification 40×).

Transiently transfected HEK293T were grown in Dulbecco’s modified Eagle’s medium (DMEM) supplemented with 1% penicillin-streptomycin, 1% nonessential amino acids, and 10% foetal bovine serum (FBS). In order to test the influence of cell density, cells were seeded on adequate support, either at a density of 1 × 10^4^ cells/cm^2^, referred to as the low-density (LD) condition, or at a density of 3 × 10^5^ cells/cm^2^, referred to as the high-density (HD) condition, corresponding to non-confluent or confluent cells, respectively, on the day of experiments, after 72 h of culture: 24 h of transfection plus 48 h of normoxia or hypoxia (Figure 1). Cells were maintained at 37 °C in a humidified atmosphere gassed with 5% CO_2_. HEK293T from control group (“normoxia”) were exposed to a gas mixture containing 21% O_2_, 74% N_2_, and 5% CO_2_. Cells from the hypoxia group were exposed to a gas mixture containing 1% O_2_, 94% N_2_, and 5% CO_2_ (“hypoxia”) for 48 h in a tri-gas incubator (Heracell 150i, ThermoScientific, Saint Herblain, France).

### 2.2. Plasmids and Transfection

To generate the BRET constructs, mNeonGreen (mNeonG) and nanoluciferase (nLuc) were mainly used to improve the brightness of the assay and are referred to as mNeonG and nLuc in the rest of this paper. For confocal microscopy, TRPV4-YFP and non-tagged TRPV4 were used in biotinylation and calcium assays, respectively in order to keep native structure and avoid signal interference of the fluorophore with the Fura emitting wavelength during the calcium test.

Mammalian expression vectors for the mNeonG-TRPV4-nLuc has been previously described in [25]. The mammalian expression vector encoding the mNeonG-Calflux-nLuc-TRPV4 fusion protein was constructed using cDNA “bricks”, obtained by gene synthesis (Genescript, Leiden, The Netherlands), that contains the cDNA sequences for mNeonG (bricks 1.1), the calflux calcium ion sensor (Calflux, brick 1.2), nLuc (brick 1.3) and TRPV4 ion channel (brick 2 and 3) as indicated in Supplementary Figure S1. The corresponding sequences are given in Supplementary Table S1.

A similar strategy was used to construct TRPV4-YFP, TRPV4-nLuc, and non-tagged TRPV4 with cDNA bricks that encode empty cDNA bricks (brick 1), to rebuild the Kozak sequence before ATG and TRPV4 ion channel (bricks 2 and 3) and YFP, nLuc, or Stop (brick 4) (Supplementary Figure S1 and Supplementary Table S1).

To construct vectors encoding mNeonG-CAAX fusion proteins, in order to evaluate TRPV4 endocytosis by measuring the decrease in the non-specific BRET (bystander BRET), we used cDNA bricks containing mNeonGreen (brick 1), and CAAX (brick 2). All brick sequences are given in Supplementary Table S1 and were provided as cDNA cloned into a pUC-Kana vector devoid of BsmBI restriction sites (Genescript).

Using the BsmB1 type IIS enzyme and T4 DNA ligase, the cDNA bricks were assembled in frame and in the right order into pcDNA3.1(+)-Lac Z vector, which allowed direct visualization of the assembly efficiency using a colorimetric test based on alpha complementation. Briefly, a ligation mix containing 2 µL of T4 DNA ligase buffer 10X, 1 U of BsmBI enzyme, 1 U of T4 DNA ligase, 0.4 mM ATP, 4 mM dithiothreitol, 200 ng of pcDNA3.1(+)-Lac Z vector, and 100 ng of each brick needed to obtain the cited final construct. Then the mix was subjected to 35–40 cycles alternating two steps (step 1: 37 °C for 1 min, step 2: 16 °C for 1 min) before the enzymes were inactivated for 5 min at 55 °C. The ligation mix was then used to transform Escherichia coli DH5α by thermal shock to amplify the plasmids.

The day of transfection, cells were trypsinized and transient transfections were performed on suspended cells using polyethylenimine (PEI, linear, Mr 25,000; Polysciences, Warrington, PA, USA) with a PEI/DNA ratio of 4:1, as already described in [26]. For patch-clamp and Ca^2+^ assays, TRPV4 WT or other BRET probes were transfected with 0.1 µg of the corresponding expression vector and 1.9 µg of empty pcDNA3.1(+) vector for a 6-well plate equivalent. For confocal imaging, TRPV4-YFP was transfected with 0.25 µg of the corresponding expression vector and 1.75 µg of empty pcDNA3.1(+) vector for a 6-well plate equivalent. When intramolecular BRET assays were performed using Calflux BRET sensor N-terminally fused to TRPV4, HEK293T cells were transfected with 0.01 µg of the corresponding expression vector and 2 µg of empty pcDNA3.1(+) vector. Alternatively, HEK293T cells were transfected with 0.01 µg of TRPV4-nLuc expression vector and 2 µg of mNeonG-CAAX expression vector for intermolecular BRET assays. Resuspended cells were seeded on adequate support (glass coverslips, Petri dishes, or 96-well plates) at low or high densities.

### 2.3. Cytoplasmic Ca^2+^ Measurements

Variations in [Ca^2+^]_c_ were determined using the Fura-2-LR/AM and Fluo-4/AM dyes. Briefly, for single cell measurements, cells seeded on glass coverslips were incubated with 5 µM Fura-2-LR/AM in Krebs-HEPES solution (see composition below) at 37 °C for 30 min, then washed and maintained in the same saline solution before fluorescence measurements. Cells were placed in a chamber of an inverted epifluorescence microscope (Nikon, Eclipse Ti2-U) equipped with a Nikon Apo Plan x40/1.3 NA oil immersion objective. Digital images were sampled at 16-bit resolution by a fast-scan digital camera (Hamamatsu, ORCA-Flash4.0 LT+). Fura-2-LR was alternately excited at 340 and 380 nm using a pE-340 Fura illuminator (CoolLED). The ratio R_340/380_ of the fluorescence intensity emitted at 510 nm at the two excitation wavelengths was calculated. Regions of interest were drawn around each cell to detect cellular ratio variations. The amplitude of the calcium rise was determined as the maximum ΔR_340/380_ reached during the recording time, i.e., between 30 and 420 s (corresponding to time after GSK1016790A application), depending on the cells. Imaging was controlled by NIS-Elements AR 5.3 imaging software (Nikon, Champigny sur Marne, France). All the images were background subtracted. These measurements allowed determining the percentage of responding cells, and then, their selection for analyses.

For cell population measurements, cells seeded in 96-well plates were incubated with 2 µM Fluo-4/AM in Krebs-HEPES solution at 37 °C for 30 min, then washed and maintained in the same saline solution before the fluorescence measurements. The Fluo-4/AM fluorescence intensity was measured at 485/520 nm (excitation/emission) using a microplate spectrophotometer reader (SPECTROstarNano2.10, BMG Labtech, Champigny sur Marne, France), in the same conditions as BRET measurements. Δ(F/F0) represents the difference between F/F0 and the basal F/F0 measured without GSK.

When mentioned, the TRPV4 inhibitor, HC067047, was added at 1 µM final concentration to the extracellular solution 30 min before the ejection of TRPV4 agonist, GSK1016790A, in the bathing solution at concentrations mentioned in the text.

### 2.4. Electrophysiological Recordings

Single-channel activity was recorded from cell-attached patches using the technique described by Hamill et al. [27]. The electrodes were pulled on a DMZ-Universal puller (Zeitz Instruments) in two stages from borosilicate glass capillaries (1.5 mm OD, 1.16 mm ID, Harvard Apparatus). The pipettes had a mean resistance of 5–7 MΩ when measured in standard recording conditions. Cells seeded on Petri dishes were viewed under phase contrast with a Nikon Diaphot inverted microscope. A RK 400 patch amplifier (Biologic) was used for cell-attached recordings. Channel activity was recorded over 60 s (one series of 60 sweeps of 1 s). Stimulus control, data acquisition, and processing were carried out on a computer fitted with a Digidata 1550 interface, using Clampex v11 software (Molecular Devices, Foster City, CA, USA). Current records were filtered with a HumSilencer noise eliminator (Molecular Devices). Data were analysed using Clampfit v11 software (Molecular Devices). GSK1016790A was applied to the recorded cell via the recording pipette in absence or presence of HC067047 in the pipette solution and bathing solution.

### 2.5. Confocal Imaging

TRPV4-YFP and mNeonG-CAAX transfected HEK293T seeded in 96-well plates were observed as “living cells” under a Nikon D-Eclipse C1 confocal scanning microscope using a Nikon Apo Plan x60/1.4 NA oil immersion objective (excitation wavelength 488 nm; emitted fluorescence recorded at 500–530 nm).

### 2.6. Cell Surface Biotinylation

Cells, seeded on 6-well plates coated with polylysine (Sigma, Saint Quentin Fallavier, France), were detached with accutase (400–600 units/mL from Sigma) and resuspended after centrifugation in ice-cold phosphate-buffered saline (PBS) supplemented with 2 mM CaCl_2_ and 2 mM MgCl_2_. Centrifugations were performed between each step to pellet cells. They were labelled for 30 min at 4 °C with membrane-impermeable EZ-link NHS-SS-biotin (0.5 mg/mL, Pierce, 21331) to biotinylate surface proteins. Afterward, the unconjugated biotin was quenched for 20 min at 4 °C with 50 mM Tris·HCl in PBS, and the cells were lysed in RIPA lysis buffer system supplemented with protease inhibitor cocktail (1:100), sodium orthovanadate (1:100), and phenylmethanesulfonyl fluoride (PMSF) (1:100) (Santa Cruz Biotechnology, Sc-24948) for 30 min at 4 °C. Insoluble materials were then removed by centrifugation at 15,000× *g* for 10 min at 4 °C, and the amount of protein was assessed by the Lowry method (Bio-Rad, 5000111, Marnes-la-Coquette, France). Equal amounts of proteins were incubated with streptavidin-agarose beads (Pierce, 20353, Rockford, IL, USA) at 4 °C overnight. The biotinylated proteins were then eluted by boiling for 10 min at 95 °C in Laemmli sample buffer (Bio-Rad, 1610747) supplemented with β-mercaptoethanol. Cell lysates of 30 μg total or of 350 μg biotinylated proteins were used for Western blot analysis.

Proteins were loaded onto a 4–20% acrylamide gel and resolved by electrophoresis. The proteins were then transferred onto nitrocellulose membranes using the Trans-Blot Turbo Transfer System. After saturation for 1 h using 0.1% TBS-Tween and 5% nonfat milk, membranes were incubated overnight at 4 °C with the primary antibody anti-TRPV4, (AB191580, 1:1000, Abcam, Paris, France). The membranes were then treated with the corresponding horseradish peroxidase-linked secondary antibodies (Pl1000, 1:10,000, Vector) for 1 h at room temperature. Membranes were processed for chemiluminescent detection using horseradish peroxidase substrate (Millipore, Molsheim, France) according to the manufacturer’s instructions. Immunoblots were then revealed and acquired using a Bio-Rad ChemiDoc XRS+ System. Images were quantified using Image Laboratory software (Bio-Rad). Equal protein loading was verified using the stain-free method.

### 2.7. BRET Measurements

#### 2.7.1. Filter-Based BRET Assays

BRET signals emitted by the total cell population in each well of 96-well plates were measured using a multidetector TriStar2 LB942 microplate reader (Berthold Technologies, Bad Wildbad, Germany) with emission filters centred at 460 ± 20 nm for nLuc (energy donor) and 510 ± 40 nm for mNeonG (energy acceptor). The BRET signal was determined by calculating the ratio of the emission intensity measured in the acceptor window (I_acceptor_) over the emission intensity measured in the donor window (I_donor_), according to “BRET” = I_acceptor_/I_donor_.

Due to the overlapping emission spectra of nLuc and mNeonG, a fraction of the light detected in the mNeonG filter originates from the nLuc emission, resulting in a contaminating signal [28]. In that configuration, the net BRET was therefore defined as the BRET ratio of cells co-expressing nLuc and mNeonG constructs minus the BRET ratio of cells expressing only the nLuc construct in the same experiment.

To assess the functionality of TRPV4 channel BRET-based probes, Furimazine, the substrate for BRET reaction, was added (diluted at 1/500 in the well) and incubated for 30 min before TRPV4 agonist was injected in the presence or absence of TRPV4 inhibitor. BRET signals were measured in DMEM w/o red phenol, 5 min after the addition of GSK1016790A, a TRPV4 agonist.

#### 2.7.2. Spectral BRET Assays

Full BRET spectra were acquired using an optical fibre linked to a spectrometer (IsoPlane SCT320, TELEDYNE, Lisse, France) equipped with a liquid-nitrogen-cooled charge-coupled device camera for recording the full visible spectrum (BLAZE 400, TELEDYNE). The bioluminescent signal was recorded from transfected cells seeded on glass coverslips and placed into an opaque home-made measurement chamber. Using the LabView programming language (National Instruments), an interface was developed to acquire the bioluminescent spectra and perform real-time spectral decomposition of the BRET signal into its various components, as described in [29].

### 2.8. Recording Solutions

The standard Krebs-HEPES extracellular solution contained (in mM): 118.4 NaCl, 4.7 KCl, 2 CaCl_2_, 1.2 MgSO_4_, 4 NaHCO_3_, 1.2 KH_2_PO_4_, 6 D-glucose, and 10 N-2-hydroxyethylpiperazine-N′-2-ethanesulfonic acid (HEPES). The osmolality (measured with a cryoosmometer type 15 Löser/Fisher Scientific, Illkirch, France) of the external salt solution was adjusted to 310 mosm/kg with mannitol, and pH adjusted to 7.4 with NaOH.

For the cell-attached patch clamp recording, the bathing solution had the following composition (in mM): 140 KCl, 2.2 CaCl_2_, 1.2 MgCl_2_, 14 D-glucose, and 10 HEPES (osmolality: 310 mosm/kg; pH = 7.4, adjusted with KOH). The recording pipette was filled with a solution containing (in mM): 140 NaCl, 2.2 CaCl_2_, 1.2 MgCl_2_, 14 D-glucose, 10 HEPES, and 10 tetraethylammonium chloride (TEA), (osmolality: 310 mosm/kg; pH = 7.4).

### 2.9. Reagents

General salts were from VWR. All other chemicals were purchased from Sigma, except FBS (Eurobio, Les Ulis, France), and furimazine which was obtained from Promega. GSK1016790A and HC067047 were dissolved in DMSO and diluted 1:2000 in the bath to the final mentioned concentrations. At this dilution, the solvent (DMSO) alone had no effect on channel activity.

### 2.10. Data and Statistical Analysis

**Figure 2:**
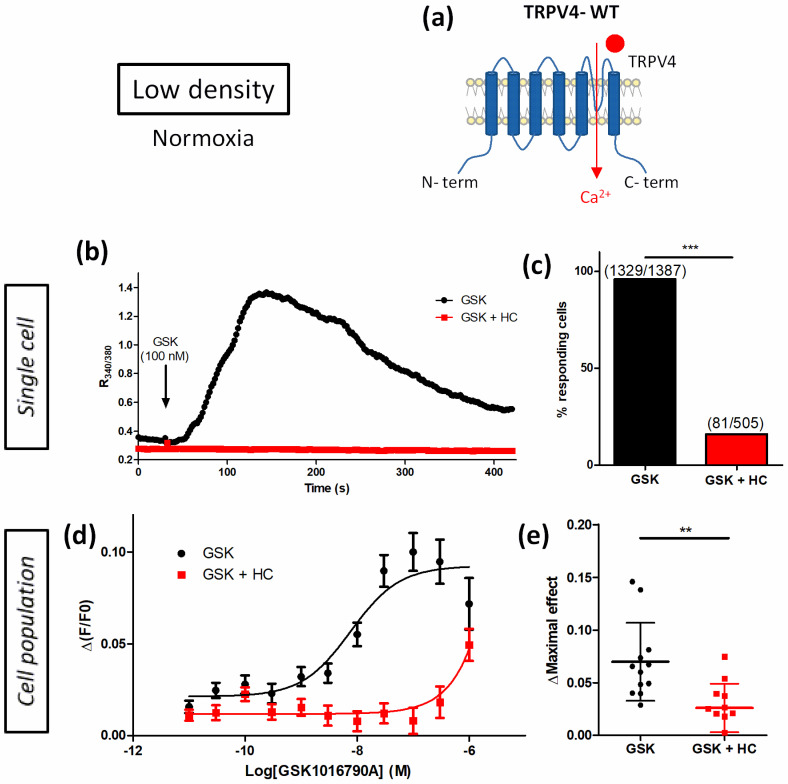
Characterization of TRPV4 Ca^2+^ response in transiently TRPV4-transfected HEK293T (HEK-TRPV4) cells cultured under normoxia at low density (1 × 10^4^ cells/cm^2^). (**a**) Schematic representation of TRPV4-WT. (**b**,**c**) Variations of relative cytosolic Ca^2+^ concentration (Ratio R_340/380_) were monitored by single-cell fluorescence videomicroscopy in Fura-2-LR/AM loaded cells. HEK-TRPV4 were bathed in physiological saline solution in absence or presence of the specific TRPV4 inhibitor HC067047 (HC, 1 μM). (**b**) Typical recordings of single cells when GSK1016790A (GSK, 100 nM), a selective TRPV4 agonist, was added at 30 s (as indicated by the arrow), and (**c**) percentage of responding cells in response to GSK. (**d**,**e**) Variations in relative cytosolic Ca^2+^ concentration (F/F0) were monitored using a microplate spectrophotometer reader in Fluo-4/AM loaded cells. (**d**) Δ(F/F0) concentration–response curves to GSK1016790A in presence or absence of HC067047 (1 µM) and (**e**) maximal concentration–response curve amplitude (ΔMaximal effect). Data are expressed as mean value ± SD. The number of cells (**c**) is indicated in brackets. Significant difference is indicated by two asterisks when *p* < 0.01 and three asterisks when *p* < 0.001, Mann–Whitney or chi-squared test.

**Figure 3:**
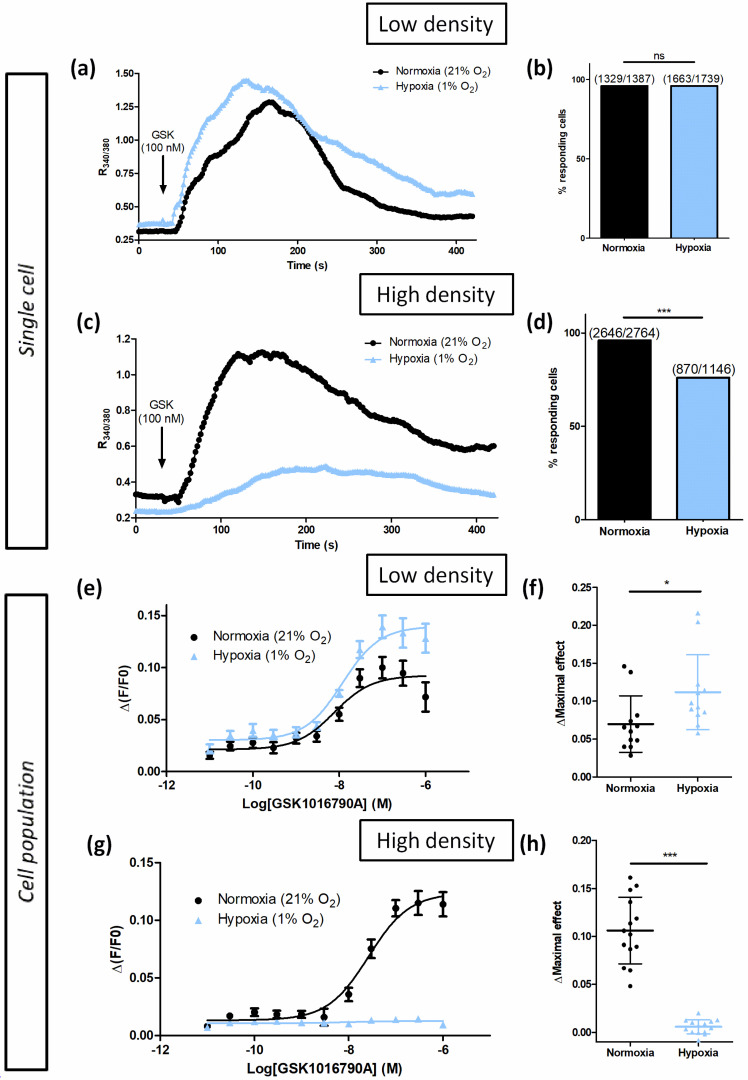
Hypoxia only potentiates TRPV4-induced Ca^2+^ responses in transiently TRPV4-transfected HEK293T (HEK-TRPV4) cells cultured at low density (1 × 10^4^ cells/cm^2^). (**a**–**d**) Variations of relative cytosolic Ca^2+^ concentration (Ratio R_340/380_) were monitored by single-cell fluorescence videomicroscopy in Fura-2-LR/AM loaded cells grown under normoxia (21% O_2_) or hypoxia (1% O_2_) for 48 h. (**a**) Typical recordings of single cells when HEK-TRPV4 were bathed in physiological saline solution and GSK1016790A (GSK, 100 nM), a selective TRPV4 agonist, was added at 30 s (as indicated by the arrow), and (**b**) percentage of responding cells in response to GSK at low density. (**c**) Typical recordings of single cells when HEK-TRPV4 were bathed in physiological saline solution and GSK1016790A (GSK, 100 nM), a selective TRPV4 agonist, was added at 30 s (as indicated by the arrow), and (**d**) percentage of responding cells in response to GSK at high density. (**e**–**h**) Variations of relative cytosolic Ca^2+^ concentration (F/F0) were monitored using a microplate spectrophotometer reader in Fluo-4/AM loaded cells grown in normoxia (21% O_2_) or hypoxia (1% O_2_) for 48 h. Δ(F/F0) concentration–response curves to GSK1016790A at (**e**) low and (**g**) high densities, and maximal concentration–response curve amplitude (ΔMaximal effect) at (**f**) low and (**h**) high densities. Data are expressed as mean value ± SD. The number of cells (**b**–**d**) is indicated in brackets. Significant difference is indicated by one asterisk when *p* < 0.05, three asterisks when *p* < 0.001, and ns indicates a non-significant difference, Mann–Whitney or chi-squared test.

Results are expressed as mean ± standard deviation (SD). The n number of responsive cells (Figure 2 and Figure 3) is indicated in each bar graph figure. A Mann–Whitney test was used to determine statistical significance between two groups. A Kruskal–Wallis followed by Dunn test was used to compare multiple groups. Comparison of cell response proportions was performed with a chi-squared test. Differences with *p* < 0.05 were considered significant. Statistical analysis was performed with the Prism 5 software (GraphPad, La Jolla, CA, USA).

## 3. Results

### 3.1. Modulation of Hypoxia-Sensitive TRPV4-Induced Ca^2+^ Response by Cell Density

#### 3.1.1. Characterization of TRPV4 Ca^2+^ Response

First, to characterize TRPV4-induced Ca^2+^ response in transiently TRPV4-transfected HEK293T (HEK-TRPV4) cells (Figure 2a), experiments were performed using its specific agonist GSK1016790A (GSK) and inhibitor HC067047 (HC) (Figure 2). When cells were cultured at low density (LD), under normoxia, in response to GSK (100 nM), the ratio R_340/380_ of the emitted fluorescence intensity, measured in single-cell Ca^2+^ imaging with Fura-2-LR/AM ratiometric probe, rapidly increased in 96% of stimulated cells (Figure 2b,c). This response was strongly inhibited in the presence of HC (1 µM) where only 16% of cells responded (Figure 2b,c) with an amplitude (ΔR_340/380_) of 0.094 ± 0.043 (n = 81 cells) instead of 1.030 ± 0.861 (n = 1329 cells) in the absence of inhibitor, respectively. In Ca^2+^ measurements with Fluo-4/AM non-ratiometric probe (cell population), TRPV4-induced Ca^2+^ response was also increased with the agonist in a concentration-dependant manner (EC50 = 8.7 ± 5.5 nM, n = 12 independent experiments). As expected, pre-treatment with 1 µM HC dramatically right-shifted the apparent potency of GSK to trigger a Ca^2+^-influx (Figure 2d,e). As already described [18], these results confirmed that GSK-induced Ca^2+^ responses were TRPV4 specific in HEK-TRPV4.

#### 3.1.2. Hypoxia Potentiates TRPV4-Induced Ca^2+^ Responses in Low-Density Cultured Cells

Previous studies have shown that prolonged hypoxia exposure occurring particularly, but not exclusively, in a variety of chronic lung diseases, increases TRPV4 activity in pulmonary arterial smooth muscle cells (PASMC) [13,14]. Moreover, a similar response was observed when PASMC were cultured under in vitro hypoxia (1% O_2_ for 48 h) [15]. We therefore tested whether this potentiating effect of hypoxia could be measured on the ectopically-expressed TRPV4 channel. For this purpose, TRPV4-transfected HEK293T cells were cultured under hypoxia (1% O_2_ for 48 h) at LD, a density commonly used for single-cell Ca^2+^ imaging studies. As expected, in hypoxic cells, the basal intracellular Ca^2+^ level was increased in single-cell Ca^2+^ imaging with a Fura-2-LR/AM ratiometric probe (Figure 3a,b). The amplitude of TRPV4-dependent Ca^2+^ response was also greater: ΔR_340/380_ = 1.053 ± 1.169 (n = 1663 cells) and ΔR_340/380_ = 1.030 ± 0.861 (n = 1329 cells) in hypoxic and normoxic cells, respectively. Accordingly, in the concentration–response curve to GSK with Fluo-4/AM non-ratiometric probe (cell population), the amplitude of TRPV4 Ca^2+^ response was increased by 61% in hypoxia without modification of EC50 (Figure 3e,f). These results show that hypoxia potentiates TRPV4 Ca^2+^ responses in HEK-TRPV4 cells cultured at LD.

#### 3.1.3. Cell Confluence Alters Hypoxia-Induced TRPV4 Ca^2+^ Response Potentiation

Although being mechanosensitive, TRPV4 modulation by cell-to-cell contact has not been investigated in previous studies. Thus, the effect of hypoxia exposure on TRPV4 Ca^2+^ response was evaluated in HEK293T cells cultured at high density (HD) to determine whether cell confluence is a determining parameter. Surprisingly, in TRPV4-transfected HEK293T cultured under hypoxia at HD, basal intracellular Ca^2+^ level was decreased in single-cell Ca^2+^ imaging with Fura-2-LR/AM ratiometric probe (Figure 3c,d). The amplitude of TRPV4 response to GSK was also smaller: ΔR_340/380_ = 0.327 ± 0.245 (n = 870 cells) and ΔR_340/380_ = 0.827 ± 0.800 (n = 2646 cells) in hypoxic and normoxic cells, respectively, as well as the percentage of responding cells. Accordingly, in concentration–response to GSK with Fluo-4/AM non-ratiometric probe (cell population), the amplitude of TRPV4 Ca^2+^ response was drastically diminished under hypoxia (Figure 3g,h). Unexpectedly, these results show that hypoxia inhibits TRPV4 Ca^2+^ responses to GSK in TRPV4-transfected HEK293T cells cultured at HD. Thus, these data draw attention to the importance of cell confluence during in vitro experiments, being a cause of altered TRPV4 Ca^2+^ response.

### 3.2. Modulation of Hypoxia-Sensitive TRPV4 Currents by Cell Density

#### 3.2.1. Hypoxia Potentiates TRPV4 Currents in Low-Density Cultured Cells

**Figure 4:**
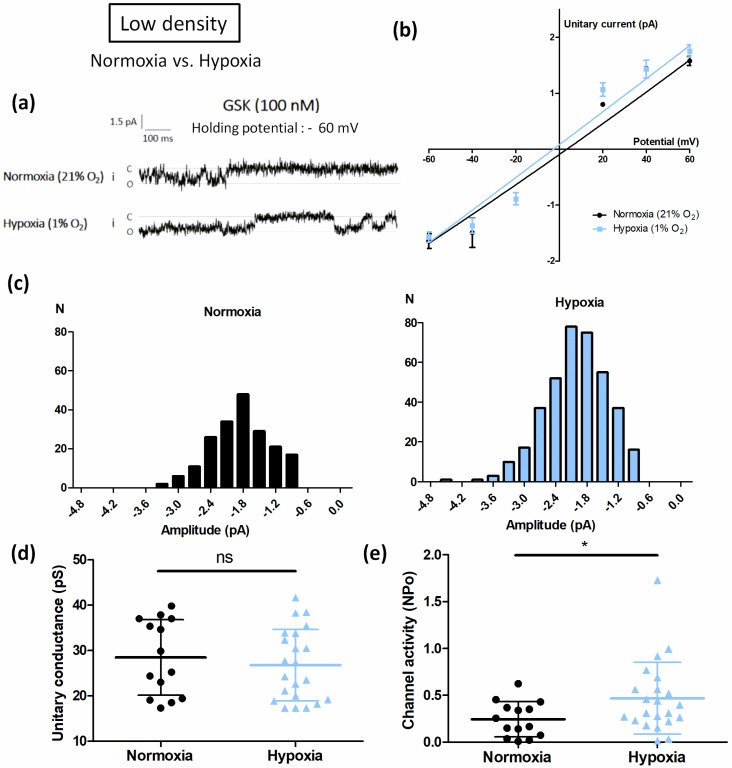
Hypoxia potentiates TRPV4 currents in transiently TRPV4-transfected HEK293T (HEK-TRPV4) cells cultured at low density (1 × 10^4^ cells/cm^2^). (**a**) Representative records of current traces (patch clamp; cell-attached configuration at −60 mV holding potential, assuming a resting potential of 0 mV) recorded in HEK-TRPV4 cells cultured under normoxia (21% O_2_) or hypoxia (1% O_2_) for 48h at low cell confluence (1 × 10^4^ cells/cm^2^). The letters c and o indicate the closed and open channel states, respectively. (**b**) Unitary current (i)/potential (V) curves, (**c**) corresponding amplitude histograms shown at −60 mV, (**d**) unitary conductance, and (**e**) channel activity (NPo), determined at a holding potential of −60 mV in presence of the TRPV4 agonist GSK1016790A (100 nM). Data are expressed as mean value ± SD. Significant difference is indicated by one asterisk when *p* < 0.05, and ns indicates a non-significant difference, Mann–Whitney test.

We then sought to confirm this first set of results by using a second technique to study the activity of the TRPV4 channel. We thus investigated the effect of in vitro exposure to hypoxia (1% O_2_ for 48 h) on single TRPV4 current using cell-attached patch-clamp recording in TRPV4-transfected HEK293T cells cultured at LD. Holding potentials were applied from null resting membrane potential obtained using high potassium (140 mM) containing bath solution. Single channel activity was evaluated in presence of GSK (100 nM) inside the patch pipette and unitary current/voltage curves were drawn for normoxic and hypoxic cells (Figure 4a,b). Unitary TRPV4 currents were then analysed at -60 mV holding potential. Conductance and open probability were determined. Whereas hypoxia did not alter TRPV4 unitary conductance (28.5 ± 8.3 pS (n = 14 cells) and 26.8 ± 7.9 pS (n = 22 cells) for normoxic and hypoxic cells, respectively (Figure 4c,d)), channel activity was increased 1.9-fold (Figure 4e). Thus, as expected, in cells cultured at LD under hypoxia, TRPV4 channel activity was potentiated by these culture conditions.

#### 3.2.2. Cell Confluence Alters Hypoxia-Induced TRPV4 Current Potentiation

**Figure 5:**
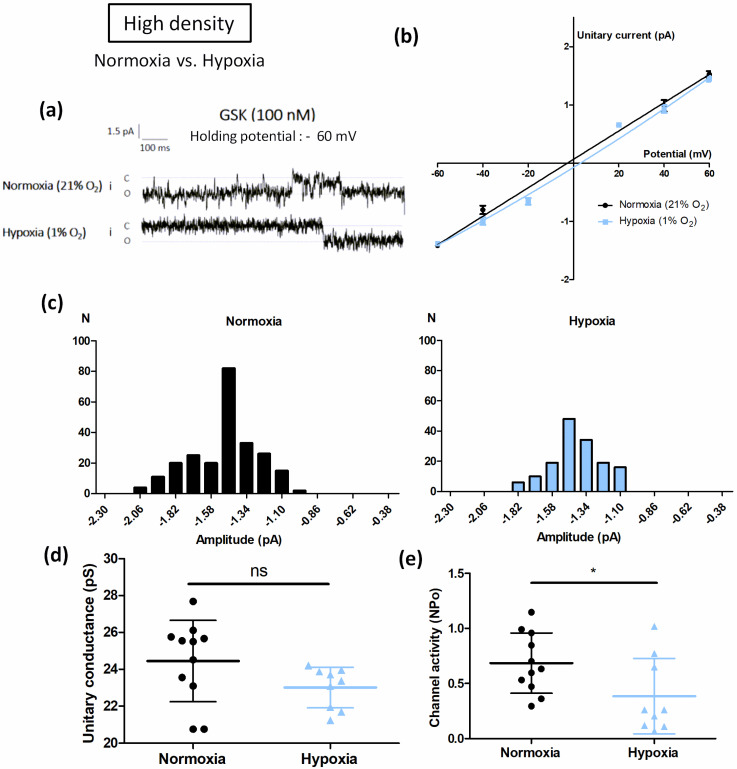
Cell confluence alters hypoxia-induced TRPV4 current potentiation in transiently TRPV4-transfected HEK293T (HEK-TRPV4) cells. (**a**) Representative records of current traces (patch clamp; cell-attached configuration at −60 mV holding potential, assuming a resting potential of 0 mV) recorded in HEK-TRPV4 cells cultured under normoxia (21% O_2_) or hypoxia (1% O_2_) for 48 h at high cell confluence (3 × 10^5^ cells/cm^2^). The letters c and o indicate the closed and open channel states, respectively. (**b**) Unitary current (i)/potential (V) curves, (**c**) corresponding amplitude histograms shown at −60 mV, (**d**) unitary conductance, and (**e**) channel activity (NPo), determined at a holding potential of −60 mV in presence of the TRPV4 agonist GSK1016790A (100 nM). Data are expressed as mean value ± SD. Significant difference is indicated by one asterisk when *p* < 0.05 and ns indicates a non-significant difference, Mann–Whitney test.

The influence of cell density on TRPV4 single channel activity was then evaluated using the same protocol with cells cultured at HD under hypoxia. Unitary current/voltage curves were drawn for normoxic and hypoxic cells (Figure 5a–c). As for cells grown at LD, TRPV4 unitary conductance was unaffected by hypoxia: 24.4 ± 2.2 pS (n = 7 cells) and 23.0 ± 1.1 pS (n = 9 cells) for normoxic and hypoxic cells, respectively (Figure 5d). Nevertheless, channel activity was decreased 0.6-fold in hypoxic cells (Figure 5e). Thus, as expected, in cells cultured at HD under hypoxia, TRPV4 channel activity was reduced by these culture conditions. These electrophysiological data corroborate those obtained for Ca^2+^ responses, confirming that cell confluence is a crucial parameter to consider.

### 3.3. Cell Density Induces TRPV4 Relocation under Hypoxia

#### 3.3.1. Cell Confluence Reduces Plasma Membrane Density of TRPV4 Channels

**Figure 6:**
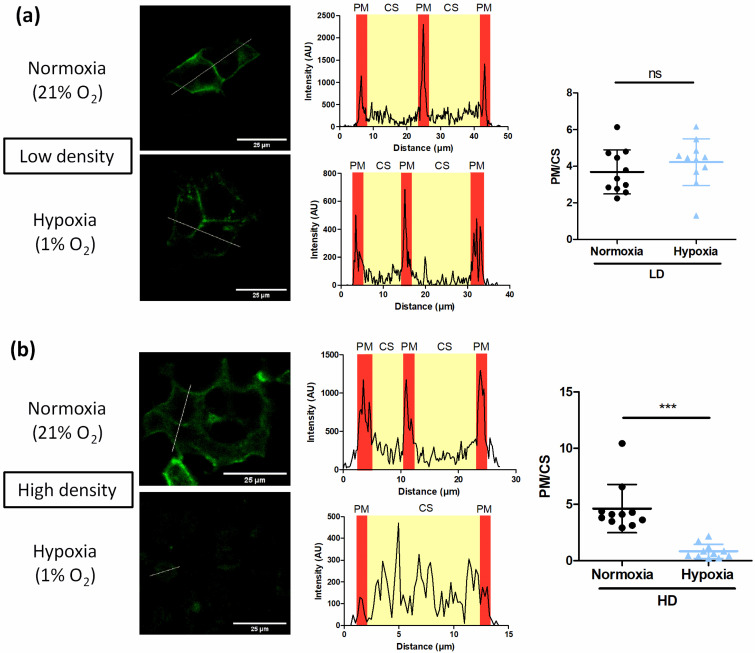
Cell confluence reduces plasma membrane density of TRPV4 channels in TRPV4-transfected HEK293T cells under hypoxia. Representative confocal fields of living HEK293T cells transfected with TRPV4 tagged with YFP and cultured at (**a**) low (1 × 10^4^ cells/cm^2^) and (**b**) high density (3 × 10^5^ cells/cm^2^) under normoxia (21% O_2_) or hypoxia (1% O_2_) for 48 h (scale bar = 25 µm); and corresponding evaluation of TRPV4 membrane localization by the fluorescence intensity profile in arbitrary units (AU) of a typical line scan indicated by the dotted line in cell. The average ratios (PM/CS) of the fluorescence signals of the plasma membrane (PM) to the fluorescence signals of the cytosol (CS) of representative cells are indicated as mean value ± SD. Significant difference is indicated by three asterisks when *p* < 0.001 and ns indicates a non-significant difference, Mann–Whitney test.

A possible explanation for this lack of response to hypoxia at HD could be a relocation of TRPV4. Thus, membrane expression of TRPV4 was evaluated using confocal imaging on living HEK293T cells expressing TRPV4 tagged with YFP, cultured at LD and HD under normoxia or hypoxia. At LD, TRPV4 channels were mainly expressed in the vicinity of the plasma membrane in both normoxic and hypoxic cells (Figure 6a). At HD, whereas TRPV4 were localized close to the plasma membrane in normoxic cells, TRPV4 channels’ locations were more diffuse in the cytoplasm of hypoxic cells (Figure 6b). Indeed, at HD, hypoxia decreased the average ratio (PM/CS) of the fluorescence signal of the plasma membrane (PM) to the fluorescence signal of the cytosol (CS) from 4.63 ± 2.15 (n = 11 fields) to 0.84 ± 0.63 (n = 11 fields) in normoxic and hypoxic cells, respectively (Figure 6b).

**Figure 7:**
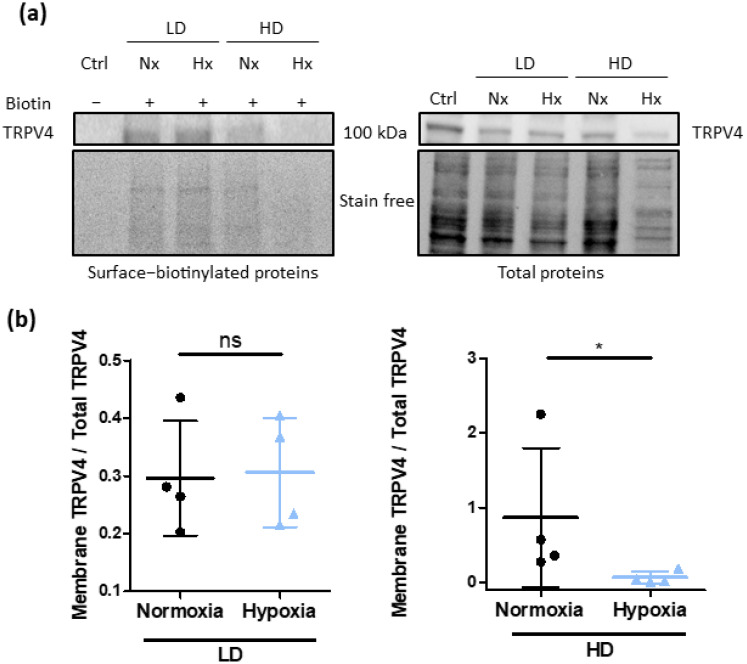
Cell confluence reduces plasma membrane expression of TRPV4 channels in TRPV4-transfected HEK293T cells under hypoxia. (**a**) Representative Western blots showing the presence of TRPV4 surface-biotinylated and total proteins from HEK293T cells transfected with TRPV4 WT and cultured at low (1 × 10^4^ cells/cm^2^) and high density (3 × 10^5^ cells/cm^2^) under normoxia (21% O_2_) or hypoxia (1% O_2_) for 48 h. Samples without biotin were used as negative control. (**b**) Membrane TRPV4/Total TRPV4 ratio was evaluated for each condition with stain free normalization. Data are expressed as mean value ± SD. Significant difference is indicated by one asterisk when *p* < 0.05 and ns indicates a non-significant difference, Mann–Whitney test.

We confirmed these observations with cell surface biotinylation of TRPV4 (Figure 7). Indeed, TRPV4 was expressed at plasma membrane in cells cultured at low density under both normoxia and hypoxia. However, less TRPV4 was detected in surface-biotinylated proteins from cells cultured at HD under hypoxia. Membrane TRPV4/Total TRPV4 ratio with stain free normalization decreased from 0.86 ± 0.93 under normoxia to 0.07 ± 0.08 under hypoxia (n = 4 independent experiments). These results confirm that cell confluence induces TRPV4 internalization in hypoxia.

#### 3.3.2. Cell Confluence Induces TRPV4 Internalization under Hypoxia

Then, membrane expression of TRPV4 was evaluated using a highly dynamic BRET- endocytosis sensor adapted from the study by Namkung et al. [24]. Briefly, the assay relies on the non-specific BRET measurement (bystander BRET) between TRPV4 C-terminally fused to the nLuc (TRPV4-nLuc) and the mNeonG fluorescent acceptor that was anchored to the plasma membrane thanks to its C-terminus fusion to the polybasic sequence and prenylation CAAX box of KRas (mNeonG-CAAX) [24,30] (Supplementary Figure S2). In this assay, the decrease in the bystander BRET between TRPV4-nLuc and mNeonG-CAAX is a direct measure of TRPV4 internalisation and loss of TRPV4 plasma membrane expression.

Since nLuc is an enzyme that consumes O_2_ in presence of furimazine, its substrate, to produce visible photons according to the reaction: Furimazine + O_2_ → Furimamide + CO_2_ + light, we first verified that the diminution of the concentration of O_2_ did not affect BRET reaction. As reported in Figure 2, no modification of the emission spectra was observed, validating the use of these BRET probes in hypoxic conditions.

Moreover, electrophysiological characterization of the BRET probes was performed to verify that TRPV4 channel properties were not modified in the presence of the nLuc. Single channel activity was recorded, and unitary current/voltage curves were drawn for normoxic cells cultured at LD (Supplementary Figure S3). No significant difference in unitary conductance and channel activity were observed between TRPV4-WT and TRPV4-nLuc (Supplementary Figure S3c,d). These results confirmed that the fusion of the nLuc to TRPV4 did not alter TRPV4 channel activity. Thus, TRPV4-nLuc probe can be used to investigate the modification of TRPV4 signalling observed in hypoxia at HD.

**Figure 8:**
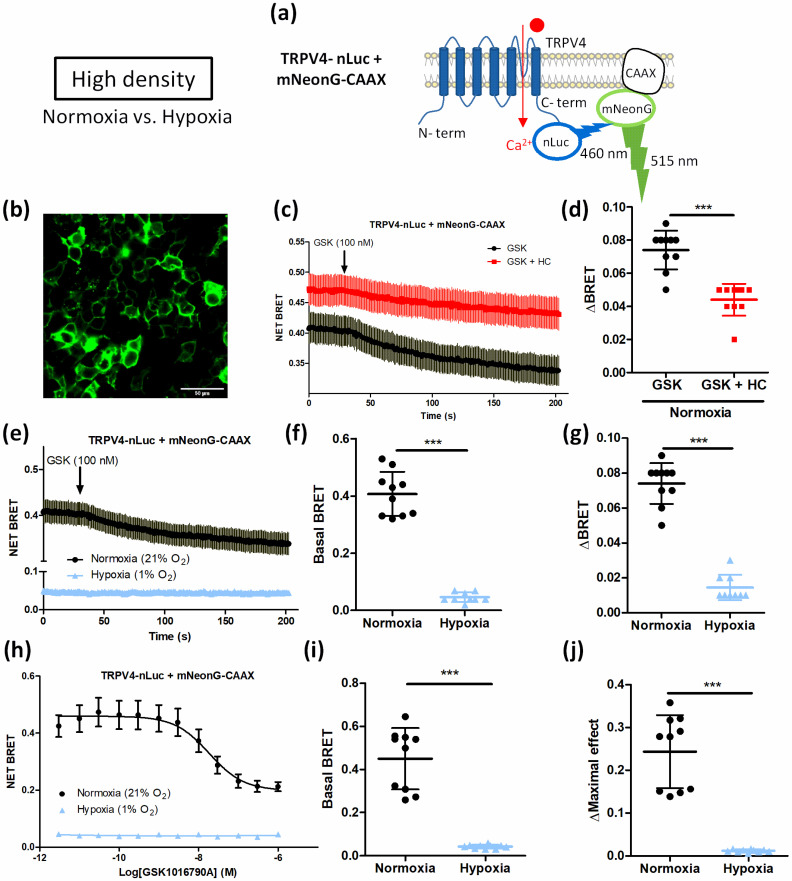
Hypoxia impairs agonist-induced TRPV4 internalization in transiently TRPV4-transfected HEK293T cells cultured at high density. (**a**) Schematic representation of TRPV4-nLuc and mNeonG-CAAX. (**b**) Representative confocal fields of mNeonG-CAAX transfected HEK293T cells (scale bar = 50 µm). (**c**) Kinetic BRET measurement of the effect of GSK1016790A (100 nM) on cells transfected with mNeonG-CAAX and TRPV4-nLuc BRET probes in presence or absence of HC067047 (1 µM) and cultured at high cell confluence (3 × 10^5^ cells/cm^2^) under normoxia, and (**d**) maximal absolute amplitude (ΔBRET). (**e**) Kinetic BRET measurement of the effect of GSK1016790A (100 nM) on HEK293T cells transfected with mNeonG-CAAX and TRPV4-nLuc BRET probes and cultured under normoxia (21% O_2_) or hypoxia (1% O_2_) for 48 h, (**f**) basal BRET, and (**g**) maximal absolute amplitude (ΔBRET). (**h**) Concentration–response curves of the effect of GSK1016790A on HEK293T cells transfected with mNeonG-CAAX and TRPV4-nLuc BRET probes and cultured under normoxia or hypoxia, (**i**) basal BRET, and (**j**) maximal concentration–response curve amplitude (ΔMaximal effect). Data are expressed as mean value ± SD. Significant difference is indicated by three asterisks when *p* < 0.001, Mann–Whitney.

BRET measurements with TRPV4-nLuc and mNeonG-CAAX (Figure 8a) showed that TRPV4 expression at the plasma membrane decreased after GSK stimulation (100 nM), with a maximal absolute BRET amplitude of 0.074 ± 0.012 (n = 10 independent experiments) (Figure 8c,d). These results confirm findings from Baratchi et al. showing that TRPV4 is internalized and translocates to the recycling endosomes after GSK stimulation [10]. In presence of the TRPV4 inhibitor (HC067047), the absolute amplitude of BRET decrease was reduced to 0.044 ± 0.010 (n = 10 independent experiments) (Figure 8c,d), suggesting less TRPV4 internalization after GSK stimulation.

When cells were cultured under hypoxia at HD, basal BRET in kinetics was highly reduced from 0.407 ± 0.077 (n = 10 independent experiments) in normoxic cells to 0.047 ± 0.017 (n = 9 independent experiments) in hypoxic cells (Figure 8e,f). Accordingly, maximal absolute BRET amplitude was 81% lower in hypoxic cells suggesting TRPV4 was already internalized before GSK stimulation (Figure 8e,g). Consistent with these findings, the concentration–response curve to GSK confirmed that basal BRET and BRET decrease in response to GSK were reduced by 91% and 95% respectively in hypoxia (Figure 8h–j). Thus, TRPV4 internalization in response to GSK stimulation was lower in hypoxia at HD due to a loss of membrane expression. This suggests that cell confluence can modulate TRPV4 addressing.

**Figure 9:**
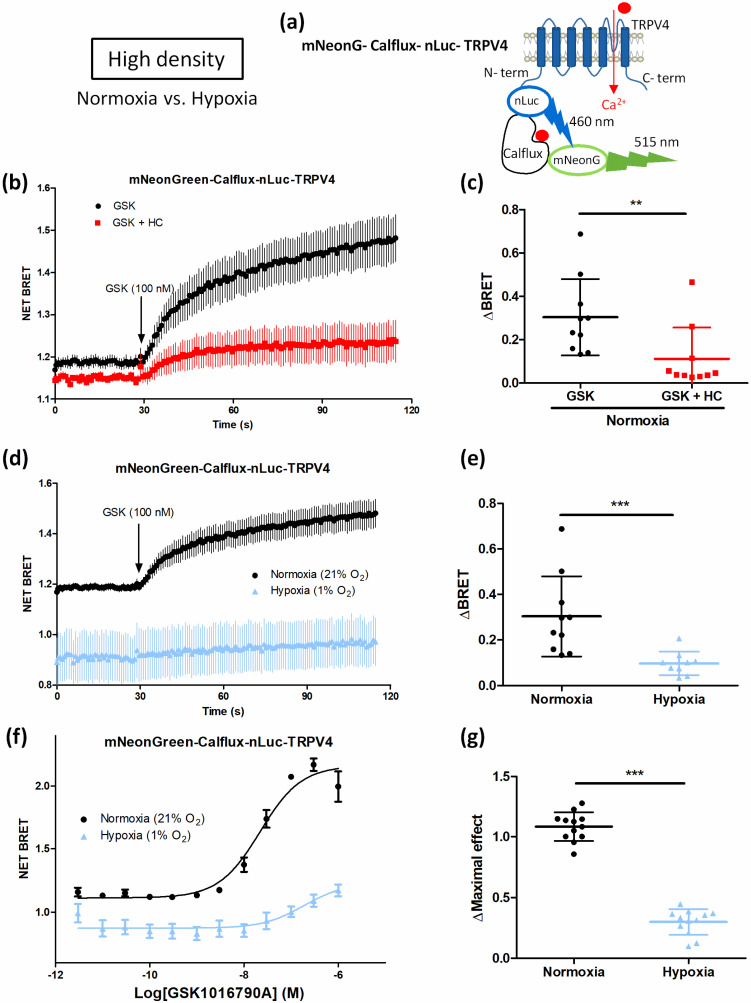
Cell confluence alters Ca^2+^ influx through TRPV4 in transiently mNeonG-Calflux-nLuc-TRPV4-transfected HEK293T cells cultured under hypoxia (1% O_2_) for 48 h at high cell confluence (3 × 10^5^ cells/cm^2^). (**a**) Schematic representation of mNeonG-Calflux-nLuc-TRPV4. (**b**) Kinetic BRET measurement of the effect of GSK1016790A (100 nM, applied as indicated by the arrow) on cells transfected with mNeonG-Calflux-nLuc-TRPV4 in presence or absence of HC067047 (1 µM) and cultured at high cell confluence (3 × 10^5^ cells/cm^2^) under normoxia (21% O_2_), and (**c**) maximal amplitude (ΔBRET). (**d**) Kinetic BRET measurement of the effect of GSK1016790A (100 nM) on cells transfected with mNeonG-Calflux-nLuc-TRPV4 cultured at high cell confluence under normoxia or hypoxia (1% O_2_) for 48 h, and (**e**) maximal amplitude (ΔBRET). (**f**) Concentration–response curves of the effect of GSK1016790A on HEK293T cells transfected with mNeonG-Calflux-nLuc-TRPV4 BRET probe and cultured under normoxia or hypoxia at HD, and (**g**) maximal concentration–response curve amplitude (ΔMaximal effect). Data are expressed as mean value ± SD. The number of independent experiments is indicated in brackets. Significant difference is indicated by two asterisks when *p* < 0.01 and three asterisks when *p* < 0.001, Mann–Whitney test.

To confirm that hypoxia impairs agonist-induced TRPV4 response because of TRPV4 internalization when HEK-TRPV4 were cultured at HD, Ca^2+^ influx in the TRPV4 pore nanoenvironment was directly monitored using the mNeonG-Calflux-nLuc-TRPV4 BRET Ca^2+^ sensor (Figure 9). Briefly, as already described for the mNeonG-Calflux-nLuc TRPV1 BRET Ca^2+^ sensor [23], the TRPV4 channel was fused to the recently described BRET sensor with high affinity for Ca^2+^ (Calflux-VTN) [31]. The troponin C domain of the Calflux-VTN BRET sensor was first sandwiched between the mNeonG fluorescent protein and the nLuc bioluminescent protein. The resulting BRET-based Ca^2+^ sensor (mNeonG- Calflux-nLuc) was then genetically fused to the N-terminal extremity of the channel.

First, electrophysiological characterization of the BRET probe was performed to verify that TRPV4 channel properties were not modified in presence of the mNeonG-Calflux-nLuc BRET probe (Supplementary Figure S3a–d). Then, HD-grown HEK293T cells expressing mNeonG-Calflux-nLuc-TRPV4 BRET probe (Figure 9a) were processed for BRET analysis to monitor Ca^2+^ influx in the TRPV4 pore nanoenvironment in both normoxic and hypoxic conditions. In absence of the TRPV4 inhibitor HC, 100 nM GSK induced a rapid and sustained BRET increase, reflecting Ca^2+^ influx through TRPV4 channel (Figure 9b,c). As expected, a diminution of 63% of BRET maximal amplitude was measured in the presence of HC (Figure 9b,c) confirming that Ca^2+^ transiting through TRPV4 channel is dependent on its activation.

When cells were cultured under hypoxia, the magnitude of the maximal response to GSK was also inhibited by 68% in response to GSK (Figure 9d,e) and the maximal efficacy of GSK was decreased by 73% in the concentration–response curve with a right-shift of the apparent potency of GSK (Figure 9f,g). These results suggest that less Ca^2+^ transits through TRPV4 in hypoxic cells grown at HD, in accordance with our previous results (Figure 3 and Figure 5). Altogether, these data show that at HD, hypoxia induces TRPV4 internalisation and thereby alters agonist-induced TRPV4 activation. These results therefore suggest that, at high cell confluence, hypoxia can influence TRPV4 activity by modulating the density of TRPV4 at the plasma membrane.

## 4. Discussion

In previous studies, in PASMC from chronically hypoxic rats naturally expressing TRPV4, we and others have already described that in vivo chronic hypoxia increases TRPV4 channel activity [13,14]. We have also shown that culturing PASMC from normoxic rats under in vitro hypoxia (1% O_2_ for 48 h) produces the same effects on TRPV4, increasing its Ca^2+^ response and channel activity [15]. In another article, the effect of hypoxia on TRPV4 was also assessed on cells without native expression of the channel [18]. In that study, TRPV4-dependent Ca^2+^ influx was reported to be increased in TRPV4-transfected HEK293T cells (cultured at 4 × 10^4^ cells/cm^2^, corresponding to LD in our study) treated with cobalt chloride, an experimental condition known to mimic hypoxia [18]. In the present study, we have shown that TRPV4-transfected HEK293T cells cultured at LD under hypoxia (1% O_2_ for 48 h) display higher TRPV4 responses to GSK stimulation.

Indeed, both Ca^2+^ responses and TRPV4 channel activity were increased in the hypoxic condition, confirming the effect of hypoxia on TRPV4 in vitro. How TRPV4 is modulated by hypoxia is still unclear, but there is evidence suggesting a specific effect of hypoxia on binding sites of the TRPV1 channel, affecting its conformation [32]. Thus, since TRPV channel members share high sequence identity (for example TRPV1 and TRPV4 share 45% sequence identity) [33], TRPV4 may also be modulated by hypoxia through conformational changes via a specific effect of hypoxia on amino acid residues. These changes could arise from post-translational modifications such as hydroxylation. Indeed, it was previously shown that TRPV3, another member of the TRPV family, was hydroxylated by FIH (factor inhibiting hypoxia inducible factors) on asparagine 242 in intracellular ankyrin repeat domains [34]. Hypoxia, inhibitors of FIH or mutation on asparagine 242 resulted in potentiation of TRPV3-mediated currents suggesting that oxygen-dependent hydroxylation can modulate ion channel activity [34].

In addition, our study reveals an impact of cell density on TRPV4 channel activity and Ca^2+^ response modulation by hypoxia. Our results show that hypoxia had a differential effect in TRPV4-transfected HEK293T cells cultured at either LD or HD. Surprisingly, agonist-induced Ca^2+^ responses and channel activity were decreased in hypoxic cells at HD. Several studies previously reported that cell density affects various physiological processes. Accordingly, it was found that confluent osteoblastic cells had a higher basal Ca^2+^ level than non-confluent ones and that Ca^2+^ responses to ionomycin, thapsigargine, ATP, and caffeine were reduced at high density [35]. This decrease was dependent on the formation of cell junctions, diminution of the inositol 1,4,5-trisphosphate (IP_3_) receptor and smooth-surfaced endoplasmic reticulum expression [35]. Additionally, in rat aortic smooth muscle cells, there is evidence that cell confluence affects β-adrenergic receptor signalling. Indeed, at HD, isoprenaline response involved both β1- and β2-adrenergic receptors, whereas at LD, the response only involved β2-adrenergic receptor and the 3′,5′-cyclic adenosine monophosphate (cAMP) rise was reduced in comparison with cells grown at HD [36]. In smooth muscle cells, the modification of this signalling pathway could alter various physiological responses such as contraction, migration or proliferation. Modulation of β-adrenergic receptor signalling by cell confluence was reported in HEK293 cells stably overexpressing β2-adrenergic receptor. According to cell density, the transduction signalling pathway selectivity of cells’ β2-adrenergic receptor ligands was different. As a consequence, some ligands induced an increase in intracellular cAMP without activating extracellular signal-regulated kinase (ERK) only in the HD condition, whereas at LD, ERK was phosphorylated following cAMP increase [37].

An important feature of cell density that can explain the modulation of cell responses is the modification of the tension in the plasma membrane with confluence inducing a change in cell mechanical behaviour. Indeed, it was shown that the rigidity of osteoblastic and fibroblastic cells was altered with culture conditions [21]. Confluent cells were stiffer than non-confluent ones and formed aligned stress fibres in response to mechanical stress, affecting cytoskeleton microstructure. Moreover, the increased formation of cell–cell junctions in confluent cells also increased the rigidity of the cytoskeleton [21]. Moreover, hypoxia was shown to induce cytoskeleton reorganization in PASMC where actin filament and intermediate filament networks were more abundant [15]. Since TRPV4 directly interacts with the cytoskeleton [38], cell contacts can impair cytoskeleton organization and reorganization in response to hypoxia, and consequently TRPV4 trafficking or conformational change. Indeed, as reported, confluence increased claudin-2 expression, a component of tight junctions, controlled by zonula occludens-1 and -2 and Rac [39]. These cell contacts also inhibited proliferation through the reduction of the formation of F-stress fibres induced by inhibition of YAP/TAZ (co-transcriptional regulators) signalling that impaired autophagosome formation [40]. This study suggests that modifications of cell confluence and matrix stiffness regulate YAP/TAZ activity, actin dynamics, and autophagy that are involved especially in proliferation and sensitivity to hypoxia via altered Hippo signalling. This pathway is regulated by AMP-activated protein kinase and mechanistic target of rapamycin (mTOR), determining cell proliferation [40,41]. The Hippo pathway is also known to be affected by hypoxia since reactive oxygen species (ROS) production induces transcriptional changes through the MST/YAP/FoxO pathway and leads to the activation of pro-apoptotic genes [41]. The fact that both cell density and matrix stiffness, as well as hypoxia, can affect cell signalling corroborates the previous assumption that TRPV4, as a mechanosensitive channel, may be particularly affected by these parameters.

Thus, the loss of TRPV4 response under hypoxia at HD could be explained by a relocation of the channel. Indeed, our present results suggest TRPV4 internalization in these conditions. First, in confocal imaging, TRPV4 was expressed in the vicinity of the plasma membrane at LD under both normoxia and hypoxia but only in normoxia for cells grown at HD. However, at HD under hypoxia, TRPV4 staining was diffuse in the cells suggesting a cytosolic expression. Moreover, cell surface biotinylation showed that TRPV4 was expressed at the plasma membrane at LD under both normoxia and hypoxia and in normoxia for cells grown at HD. But at HD under hypoxia, TRPV4 membrane addressing was reduced. To further analyse TRPV4 membrane expression, we built on a bystander BRET assay initially described to quantitatively monitor G protein-coupled receptors (GPCRs) and β-arrestin trafficking [24]. We anticipated that such an assay could specifically assess TRPV4 endocytosis by measuring the decrease in the non-specific BRET (bystander BRET) occurring in the plasma membrane plane between TRPV4 fused to the bioluminescent donor nLuc (TRPV4-nLuc) and a fluorescent acceptor fused to a plasma membrane-anchoring peptide such as the CAAX peptide [24]. As shown in Figure 9, this assay confirmed the observation of a former study [10] indicating that GSK triggers a rapid endocytosis of TRPV4, as shown by the progressive decrease in the BRET value between TRPV4-nLuc and mNeonG-CAAX following GSK challenging. Moreover, we could confirm that, in hypoxic cells grown at HD, TRPV4 expression at the plasma membrane was highly reduced and TRPV4 did not respond anymore to a further activation by GSK. This indicates that TRPV4 activation was impaired in hypoxia at HD due to an already internalized channel in this condition. The study from Baratchi et al. reported that TRPV4 translocated to the recycling endosomes after GSK stimulation and that the density of TRPV4 at the plasma membrane was controlled through both complete and partial vesicular fusion [10]. It is noteworthy that shear stress stimulation can also modify TRPV4 trafficking in endothelial cells where TRPV4 co-localized with β-catenin, an adapter protein of the adherens junctions at the point of cell–cell contact, and relocalised after shear stress, losing their interaction with β-catenin [42].

The endocytosis process is known to be mechanosensitive and can thus be regulated by membrane tension and rigidity in association with changes in membrane composition. It was shown that increased membrane tension and rigidity decrease global endocytosis [43,44] whereas hypoxia promotes endocytosis (e.g., of the Na^+^/K^+^-ATPase and alveolar epithelial sodium channel (ENaC) [44].

Overall, the combination of high cell density and hypoxia might change cell rigidity and lipid membrane composition, modulating TRPV4 activation. Such modifications could affect TRPV4 localisation through impairment of TRPV4 addressing to membrane or internalization following desensitisation of the channel. Even though our data show that TRPV4 is clearly sensitive to cell density, it is highly probable that many other channels can also be affected by cell density. Indeed, mechanosensitivity is very important in many physiological processes. A wide range of channels (SAC, stretch-activated channels) act as mechanotransducers by converting physical forces into biological signals [20]. Thus, the loss of TRPV4 expression to the membrane could be compensated by the action of other SAC. Indeed, such a compensatory mechanism was demonstrated for filamins in cultured podocytes exposed to mechanical stress where the knock-out of filamin A was compensated by the expression of filamin B [45]. A similar effect was reported in smooth muscle cells in which an increase in TRPC3 expression was observed when TRPC6 was invalidated [46].

Indeed, the present study shows how experimental conditions can be important parameters when evaluating the effect of hypoxia on TRPV4 channel, and more widely on ionic channels, considering the modification of their expression, trafficking and activity. This also raises awareness of the choice of models used in experimentation. Here, we compared cell confluence in a two dimensional (2D) cell culture model, but it would be interesting to study how TRPV4 responds to hypoxia in a three dimensional (3D) cell culture model, more relevant to physiological conditions, where cells undergo important mechanical tension since they have more cell-to-cell interactions and interact with the extracellular matrix [47,48]. Interestingly, in a 3D model, TRPV4 displayed a diffuse cytoplasmic localization in three types of cancer cells [49], suggesting that TRPV4 localisation is very sensitive to mechanical forces. In addition, another study demonstrated that osteogenic differentiation is regulated by reciprocal feedback between cell volume expansion and TRPV4 activation in mesenchymal stem cells cultured in 3D matrices [50]. These data support the idea that TRPV4 is not only sensitive to cell density in conventional 2D models but can also be affected by mechanical stress occurring in 3D models.

In conclusion, our data highlight the importance of a specific culture condition, namely cell confluence, which can influence many cellular processes. We show that HD alters TRPV4 addressing to the plasma membrane and, thus, GSK-induced Ca^2+^ responses in cells exposed to hypoxia. Accordingly, cell confluence should be taken into account when using cell culture to study various pathophysiological pathways, especially considering the differential impact of hypoxia on TRPV4 channel signalling. These results also raise awareness of the impact of techniques and methods of investigation on physiological cell responses.

## Data Availability

Not applicable.

## Supplementary Materials

The following supporting information can be downloaded at: https://www.mdpi.com/article/10.3390/biom12070954/s1, Figure S1: Schematic representation of the golden-gate strategies used to tag TRPV4 and construct the different BRET probes; Figure S2: Characterization of emission spectra of TRPV4 BRET probes in transiently transfected HEK293T cells cultured under normoxia (21% O_2_) or hypoxia (1% O_2_) at high cell confluence (3 × 10^5^ cells/cm^2^); Figure S3: Electrophysiological characterization of TRPV4 BRET probes in transiently TRPV4-transfected HEK293T cells cultured under normoxia (21% O_2_) at low cell confluence (1 × 10^4^ cells/cm^2^); Table S1: Sequences of cDNA “bricks” used.
