# Supplementary material for: Cell Confluence Modulates TRPV4 Channel Activity in Response to Hypoxia
Source: Biomolecules. 2022 Jul 7;12(7):954. doi: 10.3390/biom12070954 (PMC9313184; doi:10.3390/biom12070954)
Supplement: Supplementary file 1 [file biomolecules-12-00954-s001.zip › biomolecules-1747243-supplementary figures-Online Supplement #1 Barbeau et al (article Biomolecules) 12mai22.pdf]

Online Supplement

Cell confluence modulates TRPV4 channel activity in response to hypoxia

Solène Barbeau [1,2], Alexandre Joushomme [4,5,¶], Yann Chappe [4,5,¶], Guillaume Cardouat [1,2], Isabelle Baudrimont [1,2], Véronique Freund-Michel [1,2], Christelle Guibert [1,2], Roger Marthan [1,2,3], Patrick Berger [1,2,3], Pierre Vacher [1,2], Yann Percherancier [4,5], Jean-François Quignard [1,2] and Thomas Ducret [1,2,*]

[1] Univ. Bordeaux, Centre de Recherche Cardio-Thoracique de Bordeaux, U1045, F-33600 Pessac, France; solene.barbeau@u-bordeaux.fr, guillaume.cardouat@u-bordeaux.fr, isabelle.baudrimont@u-bordeaux.fr, veronique.michel@u-bordeaux.fr, christelle.guibert@u-bordeaux.fr, roger.marthan@u-bordeaux.fr, patrick.berger@u-bordeaux.fr, pierre.vacher@inserm.fr, jean-francois.quignard@u-bordeaux.fr, thomas.ducret@u-bordeaux.fr

[2] INSERM, Centre de Recherche Cardio-Thoracique de Bordeaux, U1045, F-33600 Pessac, France; solene.barbeau@u-bordeaux.fr, guillaume.cardouat@u-bordeaux.fr, isabelle.baudrimont@u-bordeaux.fr, veronique.michel@u-bordeaux.fr, christelle.guibert@u-bordeaux.fr, roger.marthan@u-bordeaux.fr, patrick.berger@u-bordeaux.fr, pierre.vacher@inserm.fr, jean-francois.quignard@u-bordeaux.fr, thomas.ducret@u-bordeaux.fr

[3] CHU Bordeaux, Service d'Exploration Fonctionnelle Respiratoire, F-33600 Pessac, France; roger.marthan@u-bordeaux.fr, patrick.berger@u-bordeaux.fr

[4] Univ. Bordeaux, Laboratoire de l'Intégration du Matériau au Système, UMR5518, F-33400 Talence, France; alexandre.joushomme@u-bordeaux.fr, yann.chappe@u-bordeaux.fr, yann.percherancier@u-bordeaux.fr

[5] CNRS, Laboratoire de l'Intégration du Matériau au Système, UMR5518, F-33400 Talence, France; alexandre.joushomme@u-bordeaux.fr, yann.chappe@u-bordeaux.fr, yann.percherancier@u-bordeaux.fr

* Correspondence: Centre de Recherche Cardio-Thoracique de Bordeaux, INSERM U1045, Plateforme Technologique d'Innovation Biomédicale, Hôpital Xavier Arnozan, Avenue du Haut Lévêque, F-33604 Pessac Cedex, France; thomas.ducret@u-bordeaux.fr

¶ These authors contributed equally to this work.

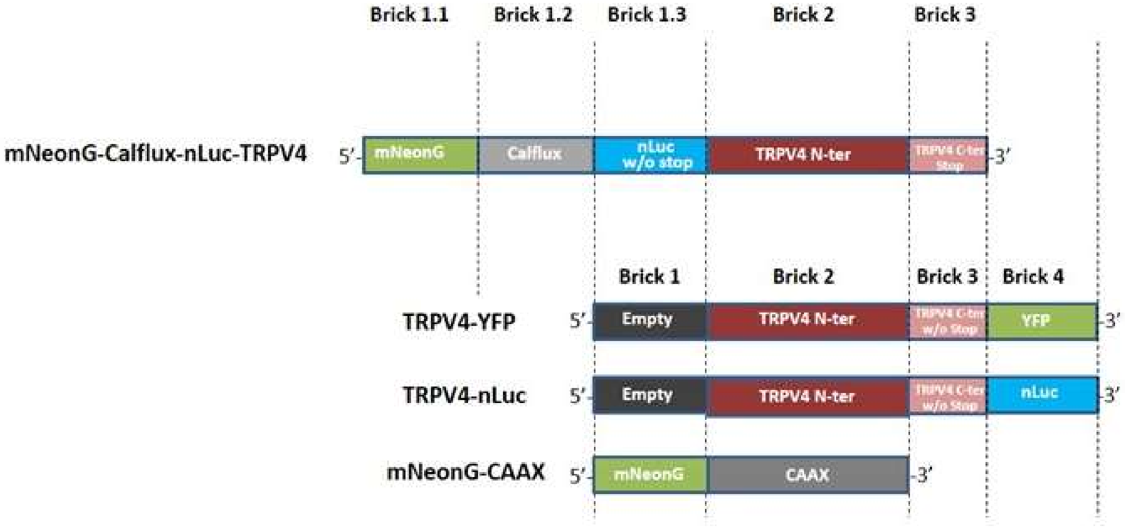

Figure S1. Schematic representation of the golden-gate strategies used to tag TRPV4 and construct the different BRET probes.

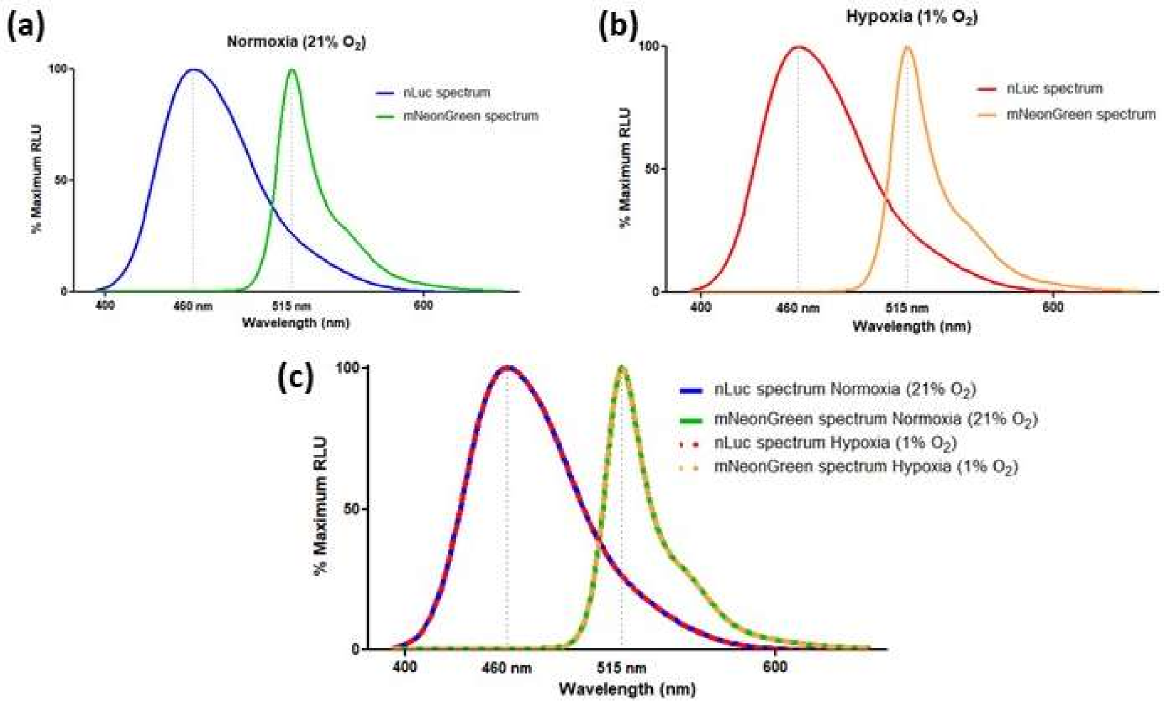

Figure S2. Characterization of emission spectra of TRPV4 BRET probes in transiently transfected HEK293T cells cultured under normoxia (21% O_2) or hypoxia (1% O_2) at high cell confluence (3×10^5 cells/cm^2). Representative emission spectra in % of maximal RLU for nLuc and mNeonG measured under **(a)** normoxia (21% O_2) and **(b)** hypoxia (1% O_2), and **(c)** merge of these spectra. RLU = Relative Light Unit.

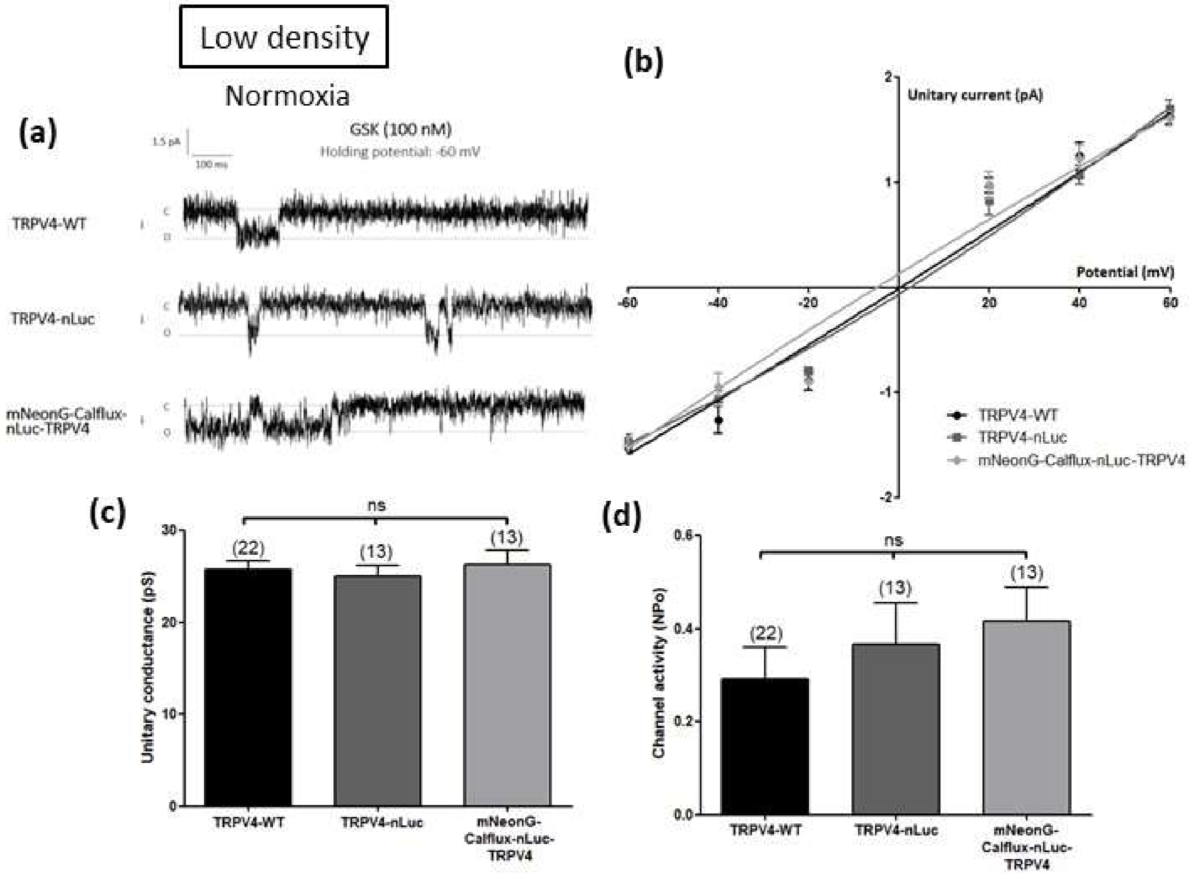

Figure S3. Electrophysiological characterization of TRPV4 BRET probes in transiently TRPV4-transfected HEK293T cells cultured under normoxia (21% O_2) at low cell confluence ($1x10^4$ cells/cm^2). **(a)** Representative records of current traces (patch clamp; cell-attached configuration at −60 mV holding potential) recorded in HEK cells transfected with TRPV4-WT, TRPV4-nLuc, and mNeonG-Calflux-nLuc-TRPV4. The letters c and o indicate the closed and open channel states, respectively. **(b)** Unitary current (i) / potential (V) curves, **(c)** unitary conductance, and **(d)** channel activity (NPo), determined at a holding potential of -60 mV in presence of the TRPV4 agonist GSK1016790A (100 nM). Data are expressed as mean value ± SEM. The number of cells (c and d) is indicated in brackets. ns indicates a non-significant difference, Kruskal-Wallis test.

Supplementary Table S1. Sequences of cDNA “bricks” used.

- ➔ cf. attached file (online supplement #2)
